# The Bleeding Duodenal Varix Dilemma: An Atypical Endoscopic Presentation of Duodenal Variceal Bleed

**DOI:** 10.14309/crj.0000000000002143

**Published:** 2026-05-22

**Authors:** Medora Doris Rodrigues, Mena Saad, Christina J. Dietz, Jonathan Scott, Christopher Bent, Wichit Srikureja, Khalid Mumtaz

**Affiliations:** 1 Division of Hospital Medicine, Department of Internal Medicine, The Ohio State University Wexner Medical Center, Columbus, OH; 2 Department of Internal Medicine, Riverside University Health System, Moreno Valley, CA; 3 Pancreas and Liver Clinic, Providence Sacred Heart Medical Center, Spokane, WA; 4 Division of Gastroenterology, Hepatology, and Nutrition, Department of Internal Medicine, The Ohio State University Wexner Medical Center, Columbus, OH

**Keywords:** duodenal varix, endoscopy, gastrointestinal bleeding

## Abstract

Cirrhosis is associated with various types of gastrointestinal bleeding, including variceal and nonvariceal. Among variceal bleeding, one of the rare causes is duodenal varices. We report a case of an atypical endoscopic presentation of a duodenal variceal bleed in a 66-year-old woman with metabolic dysfunction-associated steatohepatitis cirrhosis. Duodenal variceal bleeding can often mimic the Dieulafoy lesions, which can result in misdiagnosis, harmful interventions, and delayed treatment. This case underscores the importance on behalf of endoscopists to suspect broad differential diagnoses in patients with cirrhosis and atypical causes of gastrointestinal bleeding.

## INTRODUCTION

Cirrhosis is associated with gastrointestinal bleeding (GIB) due to variceal (70%) and nonvariceal (30%) etiologies. Among the variceal causes of GIB, most common is esophageal variceal (∼70%), followed by gastric variceal (10%–30%) bleeding, and rarely duodenal (0.4%) varices.^1,2^ Despite its rarity, duodenal variceal bleeding has a high mortality rate (up to 40%) highlighting the need for prompt diagnosis and appropriate management including endoscopic intervention.^2,3^

## CASE REPORT

**Figure 1.**
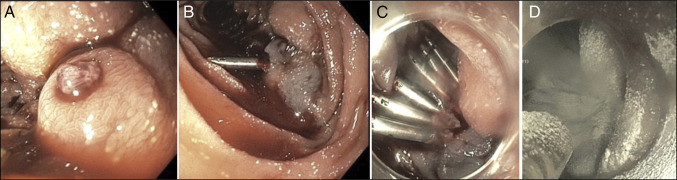
(A) Focal 4 mm submucosal bulge with a central umbilication with an embedded clot. (B) Incomplete hemostasis with epinephrine injection and singular hemostatic clip placements. Hemostasis achieved with multiple clips (C) placement and hemospray (D).

**Figure 2.**
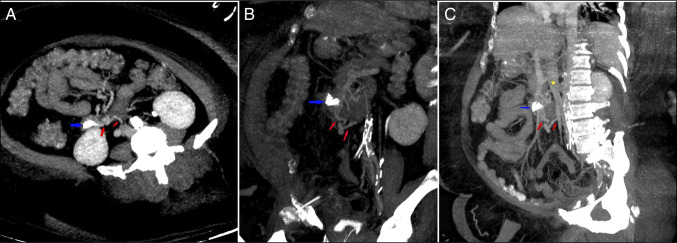
(A and B) Axial and coronal CT demonstrating endoscopic clips in the wall of the duodenum (blue arrow) with varix complex and draining outflow vein (red arrows). (C) Maximum intensity oblique coronal CT image demonstrating endoscopic clips in the wall of the duodenum (blue arrows) with varix complex and draining outflow vein (red arrows). Inferior vena cava (yellow star). CT, computed tomography.

A 66-year-old woman with class 3 obesity (body mass index: 54.8) and newly diagnosed metabolic dysfunction-associated steatohepatitis cirrhosis presented from an outside hospital with hematemesis, melena, tachycardia, and hypotension. Pertinent medical history includes type 2 diabetes mellitus, hypertension, and ovarian cancer (postsurgery and chemotherapy). She reported chronic nonsteroidal anti-inflammatory drug use, denied a previous GIB, anticoagulant use, or alcohol consumption. She was admitted to the intensive care unit. She underwent intubation, fluid resuscitation, and started on intravenous pantoprazole, octreotide, and ceftriaxone for prophylaxis as a standard of care for GIB in cirrhosis. Despite receiving 2 units of packed red blood cells and blood products, she remained hypotensive and tachycardic and started on vasopressors. Laboratory test results showed a lactate of 10, hemoglobin of 8.8, platelets of 96, total bilirubin of 3.1, albumin of 2.9, and creatinine of 0.79. Her model for end-stage liver disease score was 15. Owing to hemodynamic instability, an esophagogastroduodenoscopy was performed. Esophagogastroduodenoscopy showed no esophageal or gastric source of GIB. However, an actively bleeding lesion was found in the second part of the duodenum in the form of 4 mm submucosal bulge with central umbilication and clot (Figure 1). The lesion was initially suspected to be a duodenal ulcer or the Dieulafoy lesion. Epinephrine injection worsened bleeding despite multiple aliquots. Two preliminary metallic hemoclips were also placed but were ineffective, resulting in placement of 3 additional clips (Figure 1) with finally achieving hemostasis. Hemostatic powder was also applied (Figure 1). Her abdominal and pelvic CT showed cirrhosis and a small nonbleeding duodenal varix. The duodenal varix had an inflow from a duodenal branch of the superior mesenteric vein and outflow to the inferior vena cava near the right gonadal vein (Figure 2). While standard of care management of duodenal varix is transjugular intrahepatic portosystemic shunt or histoacryl glue, hemostasis was achieved with the hemoclips and hemostatic powder. She recovered with durable hemostasis and was subsequently extubated. The patient was later discharged in stable condition. Written informed consent was attempted to be obtained from the patient, but was unsuccessful.

## DISCUSSION

This case underscores challenges in managing ectopic duodenal variceal bleeding. Duodenal varices can mimic duodenal ulcers or the Dieulafoy lesions, leading to misdiagnosis, harmful interventions, and delayed effective treatment.^2^ In this case, the misclassification of the lesion as a Dieulafoy prompted standard hemostatic techniques including epinephrine injections and hemoclips, which not only failed, but exacerbated the bleeding. Owing to this patient not having any stigmata of liver disease, portal hypertension was ruled out as a potential differential. This was further confirmed due to the patient not having any lesions in the stomach or esophagus.

Failure of conventional therapies stress the need to maintain a broad differential in patients with cirrhosis having an atypical GIB. Other potential differentials could include liver disease, portal hypertension, and peptic ulcer disease. However, endoscopists should consider duodenal varices when encountering submucosal bulges with overlying clot and difficult hemostasis, or when the bulge has a nipple sign (Figure 1).^2–5^

While transjugular intrahepatic portosystemic shunt, histoacryl glue, or selective embolization may be curative, they are often not feasible in patients with challenging anatomy or advanced liver dysfunction. Endoscopic approaches remain the cornerstone of initial management and diagnosis.^5^ For patients with challenging anatomy, endoscopic ultrasound guided coiling with histoacryl glue or interventional radiology embolization should be considered. The successful use of hemostatic powder after failure of clips and injection highlights its important role as a salvage therapy in GIB of uncertain origin or refractory nature.^6–8^

## DISCLOSURES

Author contributions: MD Rodrigues: Study concept, design, drafting of manuscript, and final approval, and is the article guarantor. M. Saad: Study concept, design, drafting of manuscript, and final approval. CJ Dietz, J. Scott, C. Bent, W. Srikureja, and K. Mumtaz: Critical review and approval of final manuscript.

Financial disclosure: None to report.

Previous presentation: Presented at The American College of Gastroenterology Annual Meeting; October 26, 2025; Phoenix, AZ.

No Informed consent was obtained for this case report.

## ABBREVIATIONS:

CT: computed tomography
GIB: gastrointestinal bleeding
